# Study on the ownership balance and the efficiency of mixed ownership enterprises from the perspective of heterogeneous shareholders

**DOI:** 10.1371/journal.pone.0194433

**Published:** 2018-04-03

**Authors:** Zhujia Yin, Lijuan Liu, Haidong Wang, Fengming Wen

**Affiliations:** 1 School of Economics & Management, Changsha University of Science & Technology, Changsha, Hunan, China; 2 School of Economics & Trade, Hunan University, Changsha, Hunan, China; 3 Periodical Society, Central South University of Forestry and Technology, Changsha, Hunan, China; Central South University, CHINA

## Abstract

Based on the database data of Chinese industrial enterprises from 2000 to 2007 and the LP method, this paper measures the total factor productivity of enterprises and investigates the effect of different mixed ownership forms on enterprises’ efficiency and the effect of heterogeneous ownership balance on the mixed ownership enterprises’ efficiency. The state-owned enterprise and mixed ownership enterprise are identified by the enterprise’s paid-up capital. The results show that, on the whole, for the mixed ownership enterprise, the higher the diversification degree of the shareholders is, the higher the efficiency becomes, and in different types of industries, the mixed forms of shareholders have different effects on the efficiency of enterprises. The heterogeneous ownership balance and the enterprise efficiency show nonlinear U-type relationships. Both the higher and lower heterogeneous ownership balance degrees will promote the enterprise’s efficiency. However, when the ownership balance degree is in the range of [0.2 0.5], the increase in ownership balance will lead to the decline of enterprise efficiency. Therefore, when introducing non-state-owned capital, state-owned enterprises should take full account of their own characteristics by rationally controlling the shareholding ratio of non-state-owned capital and play the positive role of a mixed ownership structure in corporate governance with appropriate ownership balances.

## Introduction

The decision of the Central Committee of the Communist Party of China on several major issues concerning comprehensively deepening reform in the third Plenary Session of the 18^th^ CPC Central Committee clearly noted that the development of a mixed ownership economy—an important form of realization of China's basic economic system—showed the direction and target for the reform of state-owned enterprises. As the micro form of the mixed ownership economy, the mixed ownership enterprises refer to enterprises with cross-shareholding and the integration of state-owned and non-state-owned capital [1].

Compared with state-owned enterprises, mixed ownership enterprises have a special ownership structure, in which state-owned shareholders and non-state shareholders simultaneously exist. In regard to the mixed ownership reform of state-owned enterprises, it is necessary to make it clear whether mixed ownership enterprises are a more efficient form of enterprise organization compared with state-owned enterprises. A sizeable literature has analyzed the advantages of mixed ownership enterprises theoretically, but there is not much empirical evidence concerning mixed ownership enterprises. First, on the basis of the data of Chinese industrial enterprises, this paper studies the difference in efficiency between mixed ownership enterprises and state-owned enterprises as well as the reasons behind it and then analyzes the effect of heterogeneous large shareholders in mixed ownership enterprises on the mixed ownership enterprise’s efficiency and discusses whether there is an optimal proportion between the two types of shareholders. The empirical results show that the higher the diversification degree of the shareholders is, the higher the efficiency becomes, and the mixed forms of shareholders have different effects on the efficiency of enterprises in different types of industries. There is a U-type relationship between heterogeneous ownership balance degree and enterprise efficiency in mixed ownership enterprises. With the weakness of the heterogeneous ownership balance, the efficiency of enterprises first decreases and then rises. This study has a positive practical significance for the evaluation of mixed ownership reform and the exploration of the optimal ownership structure of mixed ownership enterprises.

The article is arranged as follows: the first part is a literature review and introduces the contributions of this paper. The second part is the theoretical analysis as well as the corresponding research hypothesis in combination with theory. The third part is the data and sample characteristics, which introduce the data source and the sample of the study. The fourth part is the empirical test of the influence of the ownership structure on enterprise efficiency. The fifth part is the empirical test of the effect of the heterogeneous ownership balance on the mixed ownership enterprise’s efficiency. The sixth part summarizes the full text and introduces policy recommendations.

## Literature review

Academic circles have paid much attention to the efficiency of state-owned enterprises for a long time, and most of the existing research on the efficiency of state-owned enterprises is based on the nature of the ownership of enterprises, conducting a comparative study on the efficiency between various types of enterprises or discussing the effect of the capital nature on the enterprise efficiency. The differences in efficiency between state-owned enterprises and other types of enterprises can be broadly divided into three categories: ① the inefficient theory of state owned enterprises. Liu finds that state capital has an obvious negative effect on efficiency [2]. Hu, Song & Zhang compare the per capita profit level and the per capita sales income and other indicators of the enterprises before privatization with the profit rate and believe that private shares and foreign shares have a more positive impact on corporate performance than state-owned shares [3]. Kong, Dai & Li also find that the production efficiency of state-owned enterprises is lower than that of foreign enterprises [4]. Wu notes that state-owned enterprises suffer not only from the loss of production efficiency but also from the loss of innovation efficiency [5]. Huang, Bai & Tan have similar findings [6]. Based on the DEA method, Fan, Yu & Zhao measure the comprehensive efficiency of state-owned, private and foreign enterprises in each province and find that the average efficiency of foreign and private enterprises was always higher than that of state-owned enterprises [7]. Abramov, Radygin, Entov & Chernova use 114 of the largest Russian companies as samples, and find that state-owned enterprises were worse than private enterprises on average [8]. ② The effective theory of state-owned enterprises. Li & Qiao use the industrial data from 1999 to 2006 and find that the overall performance of state-owned enterprises has improved significantly since the series of reforms in 1999, and the economic performance also showed signs of improvement in approximately 2003 [9]. Based on the relevant data of the Chinese industrial industry from 2003 to 2008, Ma uses the Malmquist index to calculate the total factor productivity of state-owned enterprises and foreign enterprises. It is found that the higher total factor productivity of state-owned enterprises is due to the higher rate of technological progress [10]. ③ There is no efficiency difference between state-owned enterprises and non-state-owned enterprises. Hong & Dong separately calculate the total factor productivity of state-owned and non-state-owned enterprises and finds that the efficiencies of these two enterprises have no significant difference through comparative studies [11]. Zhang & Zhang study the efficiency of state-owned and non-state-owned enterprises from the perspective of industry characteristics and find that there is no significant difference between both financial efficiency and technical efficiency in competitive industries [12]. By adopting an entropy weight evaluation method, Hao, Tian & Tao compare state-owned enterprises with private enterprises, foreign enterprises and Hong Kong-Macao-Taiwan invested enterprises and find that the efficiency of enterprises has no certain relevance to their ownership [13]. Arocenan & Oliveros compare the efficiency of Spain's pre-privatisation state-owned enterprises with competing private ones and the results show that there is no obvious difference between the efficiency of state-owned enterprises and private enterprises [14].

In the existing literature, there has not been much research on mixed ownership enterprises. Chen & Tang study the policy burden of enterprises that underwent mixed ownership reform and defined the enterprises that mixed with collective capital; personal capital; corporate capital; Hong Kong, Macao and Taiwan capital; foreign capital; and other non-state capital as mixed-ownership enterprises. The study finds that enterprises that have undergone mixed ownership reform experience a larger decline range in policy burden than enterprises that have not, so the efficiency is less affected by the policy burden [15]. Dong, Hong & Yang take the industrial enterprise data from 2005 to 2007 as the sample, and the empirical evidence shows that mixed ownership enterprises exhibit stronger solvency and better technological innovative capabilities, and the performance of a mixed-ownership economy is better than that of state-owned enterprises but worse than that of private-owned enterprises [16]. Wu & Zong take the industrial enterprise data from 1998 to 2007 as the sample and discover that the more diversified the ownership is, the more obvious the efficiency advantage of the enterprise becomes [17]. Huang, Li & Yin find that the different models of mixed-ownership enterprises have different effects on corporate performance. The equity mixed mode, in which the gap between the proportion of major state-owned shareholders and major non-state shareholders is moderate, will be more conducive to enhancing corporate performance [18]. Janang, Suhaimi & Salamudin analyze an unbalanced panel dataset of 31 government-linked companies listed at Malaysia’s Stock Exchange between 2001 and 2012 and find that there is a positive correlation between the proportion of state-owned shares and the production efficiency. However, the high concentration of ownership tends to lead to the inefficiency of the enterprises [19]. Chang & Boontham find that there is an inverted U-shaped nonlinear relationship between the annual average rate of state-owned shares and firm performance in mixed-ownership enterprises, that is, the proportion of state-owned shares must be kept within a certain range to promote the improvement of firm performance [20].

The relationship between ownership balance and enterprise efficiency has always been the focus of scholars. The existing research can be broadly divided into three categories. The first is the useful theory of ownership balance. Chen & Wang take the data of A-share listed company as samples and find that companies with ownership balance have higher corporate values than other companies [21]. Tong & Chen find that for the enterprises in the mature period, the value of the enterprises with ownership balance is higher than that of “the only big shareholder” enterprises [22]. Qiu, Shen, Liu & Zhou find that ownership balance can weaken the controlling shareholder's ability to acquire private benefits of control and thus enhance the efficiency of the company [23]. Gutierrez & Pombo test 233 non-financial listed companies in Colombia from 1996 to 2004 and find that a more equitable equity allocation among shareholders had a positive impact on the value of the company [24]. The second is that ownership balance have no significant effect on enterprises’efficiency and even have a negative effect on corporate performance. Nguyen, Rahman & Zhao find that companies with more balanced ownership structures performed better [25]. The second is that ownership balance have no significant effect on enterprises’ efficiency and even have a negative effect on corporate performance. Xu, Xin & Chen find that the excessive ownership balance degree has a negative impact on the company's operating performance and that the effect of different kinds of external big shareholders is obviously different in the listed companies with different controlling shareholders [26]. Zhu & Wang take Hongzhi technology’s equity dispute as an example and find that for private listed companies, ownership balance structure is not more efficient than a single ownership structure, and it cannot improve corporate governance efficiency [27]. Third, there is a non-linear relationship between ownership balance and enterprise efficiency. Taking the data of listed companies as samples, Wu & Wu study the relationship between ownership balance and corporate performance. The results show that the company's return on net assets has a U-type relationship with the ownership balance degree [28]. Considering the balances between the major shareholders, Liu, Lu & Song conclude the ownership balance degree and find that it has an inverted U-type relationship with the company value [29]. However, Huang,Yang & Yin, by using the new calculation method of ownership balance degree, find that ownership balance degree and the corporate performance have a U-type relationship [30].

On the whole, most studies have focused on the difference in the number or proportion of shareholdings between shareholders. However, only considering the differences in the number of shareholder holdings is not enough to reflect the impact of ownership balance on the enterprise efficiency, and the relationship between the differences in the nature of shareholders and the enterprise efficiency should also be considered.

## Theoretical analysis

Mixed ownership enterprises are the integration of state-owned capital with private and foreign capital. The advantages of state-owned capital are more government resources, higher credit rating and strong financing capacity. Its insufficiency is reflected in the fact that non-separation between government and enterprises results in an inability to have independent decision-making, and enterprises dispensing with full financial responsibility will lead to budget soft constraints and less efficient behavior alienation. The advantages of domestic private capital are flexible mechanism, independent decision-making, high efficiency, sensitivity to the market, rigid constraints, cost control and perfect management; its shortcomings are that strategy and employing aspect are not stable enough, and the strength of funds and talents is weak. Foreign capital has the advantage of relatively high technology, rich management experience, international vision, brand, sales network, business model, global technology and market development ability. The deficiency is not being familiar with the Chinese market and rules [31][32].

As a result of the introduction of the different natures of investors, mixed ownership enterprises tend to form a diversified ownership structure and a multiple interests structure. Shareholders of different natures with concerns for their own rights and interests will form an ownership balance mechanism. Shareholders have the motivation to exercise their own supervision and prevent other shareholders from obtaining discretionary control of private interests. Additionally, large shareholders can be forced to make practical decisions that can enhance the value of the company and the interests of investors to achieve complementary advantages, harmonious coexistence and integration of state-owned capital and private capital, which will ultimately improve the efficiency of enterprise management [33]. Therefore, to a certain extent, the introduction of private capital by the state-owned enterprises, through the reform of the mixed ownership system, can solve the problem of inadequate supervision within the enterprise caused by the absence of the owner of state-owned enterprises. Therefore, the first hypothesis we propose is that the introduction of non-public capital can enhance the efficiency of enterprises, making the mixed ownership of enterprises more efficient than state-owned enterprises.

However, a number of studies have also found that the simple mixing of two types of equity does not improve corporate performance [34][35]. Both state-owned and non-state capital have their own advantages and disadvantages, according to the existing research results. There is no conclusion in terms of what type of proportion and mode of mixing is best. Ma, Wang & Zhang take the state-owned competitive listed companies in the Shanghai Stock Exchange as samples and find that non-state-owned shareholders that are among the top 10 shareholders at a proportion of 30%-40% will realize the highest corporate performance [36]. Guo & Ma conduct empirical research on the data of industrial enterprises and find that the ratio of non-state-owned shares and enterprise TFP shows an "inverted U-type" relationship [37]. Huang, Li & Yin find that the ownership mixed model with a moderate shareholding ratio (10%-30%) of major state-owned shareholders and large non-state-owned shareholders is most conducive to enhancing corporate performance [18]. Wen, Xiao, Huang & Xia find the similar results [38].

The existing research failed to reach a consensus on the influence of the ownership structure on enterprise efficiency. On the one hand, it may be the difference between the sample and the calculation method of the ownership balance. On the other hand, the ownership structure has a two-way influence on enterprise efficiency itself. Particularly with the further consideration of shareholder heterogeneity, the influence mechanism becomes more complicated. In view of this, this paper uses the absolute value of the difference between the shareholding ratios of the two types of shareholders (state-owned and non-state-owned) to determine the ownership balance degree in mixed ownership enterprises and studies the relationship between the balance degree of heterogeneous shareholders and enterprise efficiency.

Based on the above analysis, our second hypothesis is that there is a non-linear relationship between the balance degree of heterogeneous shareholders and enterprise efficiency.

## Design of empirical research

### Empirical thinking

According to the needs of the research, this paper conducts the measurement test mainly from the following two levels: ①From the perspective of the ownership structure, we study the differences in the efficiency between mixed ownership enterprises and state-owned enterprises and observe the differences in the performance of different industries. ②Taking mixed ownership enterprises as the object, this paper studies the nonlinear effects of the balance between state-owned shareholders and non-state shareholders on enterprise efficiency to investigate whether there is any suitable ownership balance.

### The sample, variable and model

In the study of enterprise efficiency, corporate governance and other related content, many scholars often use listed company data, but listed company data also has its limitations. The sample size is limited and only provides information on the shareholding of the top ten shareholders, which may be biased with the company's overall ownership structure. This section takes enterprise data in Chinese industrial enterprise database as the research object. The total output value of enterprises accounted for more than 90% of China's total industrial output value in the database. The sample size is large and more universal and provides the paid-in capital of the enterprise, which can reflect the ownership structure of the enterprise more comprehensively and clearly.

The observation period is from 2000 to 2007 because there is a lack of key factors after 2008 to calculate the total factor productivity, for instance, the data of industrial value, total industrial investment and net value of fixed assets. The research sample selection and data processing refer to Nie, Jiang & Yang.: ①Exclude the samples that lack total assets, a gross industrial output value, the number of employees, intermediate industrial input or other key indicators; ②Exclude the samples that do not meet the accounting standards, in which the total assets are less than the current assets, the cumulative depreciation is less than the current year depreciation, the industrial output value is less than the industrial added value, total assets or net fixed assets is negative and so on; ③Exclude the samples where the number of employees is less than 8 and thus lack a reliable accounting system; ④Exclude the samples that do not meet the "above scale" standard, that is, enterprises with a sales volume below 5 million [39].

As a type of ownership arrangement mode, ownership balance is the way to share control rights among any big shareholder who cannot make enterprise decisions by constraints between shareholders. The academic community generally uses the proportion of the second to the tenth largest shareholder's shareholding to the proportion of the largest shareholder's shareholding or the proportion of the second to fifth largest shareholder's shareholding to the proportion of the largest shareholder's shareholding to measure the strength of the equity checks and balances. Based on the traditional ownership balance, heterogeneous ownership balance divides the shareholders into state-owned shareholders and non-state shareholders by adding to the recognition of the nature of shareholders, and then considers the mutual containment between the two types of shareholders. At present, there is not much research on ownership balance between heterogeneous shareholders and there is no uniform index for the relevant proxy variables in academia. The data used in this paper are from the Database of Chinese state-owned enterprises, which is different from the data of listed companies, so this study cannot define the ownership balance degree according to the methods of previous literature because it does not have such indexes as the proportion of the top ten shareholders. However, the database contains detailed information about the paid-in capital of the enterprise, which includes information of state-owned capital; collective capital; personal capital; corporate capital; Hong Kong, Macao and Taiwan capital; and foreign capital. Therefore, this paper takes the absolute value (| A-B |) of the difference between the ratio of state-owned shareholders' shareholding and non-state-owned shareholders' shareholding to measure heterogeneous ownership balance degree in mixed ownership enterprises. The greater the value is, the greater the difference between the two types of shareholder holdings is, and the weaker the balance between them becomes; conversely, the smaller the value indicating that the closer the shareholding ratio of the two types of shareholder is, the stronger the degree of heterogeneous ownership balance is.

In this paper, the total factor productivity (TFP) is used to measure the efficiency of enterprises; it usually refers to that part of the output production that cannot be attributed to tangible inputs, which reflects the unit average output level of the various inputs in the production process, that is, the overall efficiency from inputs into final outputs, and this paper uses the LP method to measure the TFP level rather than the TFP growth rate. The LP method is a method for estimating TFP proposed by Levinsohn & Petrin, which uses intermediate inputs as proxies but not investment, arguing that intermediates may respond more smoothly to productivity shocks. Further more, the data of intermediate product as input index is more easily to get than the amount of investment [40]. In addition, Petrin, Poi & Levinsohn reviews this approach and introduces a Stata command that implements it to expand the proxy choice set greatly. The LP method makes researchers to select proxy flexibly according to the data features [41].

To study the efficiency difference between the mixed ownership enterprises in different forms and state-owned enterprises, this paper constructs model (1):

$$TFP=c_{0}+aown+\sum b_{i}{control}_{i}+\varepsilon_{0}$$ (1)

To study the nonlinear effect of heterogeneous ownership balances on mixed ownership enterprises, this paper constructs models (2), (3) and (4):

$$TFP=c_{0}+a_{1}|AFP|+\sum b_{i}{control}_{i}+\varepsilon_{0}$$ (2)

$$TFP=c_{0}++a_{2}{\left|A+P\right|}^{2}+\sum b_{i}{control}_{i}+\varepsilon_{0}$$ (3)

$$TFP=c_{0}+a_{1}|AFP|+a_{2}{\left|AFP\right|}^{2}+\sum b_{i}{control}_{i}+\varepsilon_{0}$$ (4)

In the above regression models, as an explained variable, TFP is the total factor productivity level measured by the LP method, which is used to measure enterprise efficiency. The explanatory variables are the mixed form dummy variables, taking state-owned enterprises as the reference group and comparing that group with the mixed ownership enterprises in the form of three types of ownership ("state-owned + private", "state-owned + foreign" and "state-owned + private + foreign"). The A and B in |A-B| are the proportion of state-owned shareholders holdings and non-state-owned shareholders holdings, and the absolute value of the difference of them (|A-B|) is the heterogeneous ownership balance degree. The meaning of other control variables is shown in Table 1. The descriptive statistics of the main variables are shown in Table 2. It can be seen that the values of each variable are in a reasonable range.

**Table 1:** Description of variables.

Variable name	Variable symbol	Definition
**Dependent variable, enterprise efficiency**	*TFP*	Total factor productivity of enterprises based on LP method
**Observed variable mixed form**	*own*	The state-owned enterprises are the reference group, and the mixed ownership enterprises are the experimental group
**Heterogeneous ownership balance degree**	*|A-B|*	The absolute value of the difference between the proportion of state-owned shareholders and non-state-owned shareholders
**The square of heterogeneous ownership balance degree**	*|A-B|*^*2*^	The square of the absolute value of the difference between the proportion of the state-owned shareholders and the proportion of non-state shareholders
**Control variable, enterprise scale**	*size*	The numerical value of the total assets
**Enterprise age**	*age*	Year of establishment of enterprise
**Asset liability ratio**	*lev*	The ratio of total liabilities to total assets
**Fixed assets ratio**	*fae*	The ratio of fixed assets to total assets
**Subsidy rate**	*sub*	The ratio of subsidized income to sales revenue
**Industry**	*ind*	40 sectors of the national economy
**Province**	*pro*	31 provincial administrative regions of the country (excluding Hong Kong, Macao and Taiwan)
**Years**	*year*	From 2000 to 2007

**Table 2:** Descriptive statistics of the main variables.

Variable name	Number of samples	Mean value	Standard deviation	Minimum value	Maximum value
**TFP**	259835	5.889	1.063	-2.994	14.091
**|A-B|**	259835	0.773	0.328	0	1
**|A-B|**^**2**^	259835	0.705	0.394	0	1
**size**	259835	10.244	1.556	3.296	18.856
**age**	259835	17.432	13.683	1	59
**lev**	259835	0.630	0.343	0	20.049
**fae**	259835	0.379	0.225	0.000	1.318
**sub**	259835	0.007	0.038	0	7.433

## The influence of mixed ownership structure on enterprise efficiency

To examine the efficiency difference of the ownership structure, the state-owned enterprises are taken as the reference group, and the mixed ownership enterprises in the form of three types of ownership ("state-owned + private", "state-owned + foreign" and "state-owned + private + foreign") are taken as the experimental group. In addition to the full sample regression, the sample is divided into labor intensive industries, capital intensive industries and technology intensive industries according to the nature of the industry.

The Hausman test [42] shows that the panel data fixed effect model is more suitable for the model in this paper. In the regression results of Table 3, the first three explanatory variables are mixed ownership variables, whose coefficients are positive, indicating that the enterprises in this form are more efficient than the state-owned enterprises. According to the results, the efficiency of three types of mixed-ownership enterprises is significantly higher than that of the state-owned enterprises in the sample, which is consistent with the conclusion of Wu [17]. Among them, the coefficient of "state-owned + private + foreign" is the largest (0.067), the coefficient of "state-owned + foreign" is in the middle (0.028), and the coefficient of "state-owned + private" is the smallest (0.015). Then, by observing the results of group regression, it can be found that the estimation coefficients of the control variables in the group regression results are in agreement with the results of the full sample regression. The estimation coefficients of most mixed ownership variables are also consistent with the results of the full sample regression, so the robustness of the model is verified. By analyzing the coefficients of mixed ownership variables in the regression results, the following conclusions can be drawn: ①In the labor intensive industry group, the mixed ownership enterprises in the form of "state-owned + private + foreign" is more efficient than state-owned enterprises, and the coefficient is smaller than the full sample, which means that the effect of "mixed reform" on labor intensive industries is not good. The reason may be that the labor intensive industries use the labor force extensively, the dependence on technology and equipment is relatively low, and it is difficult to capitalize on the technological innovation advantage of non-state capital in this type of industry. ②In the capital intensive industry group, the impact of the mixed ownership enterprises in the form of "state-owned + foreign" on enterprise efficiency is not significant, and the mixed ownership enterprises in the two forms of "state-owned + private" and "state-owned + private + foreign capital" are significantly better than state-owned enterprises; it can be inferred that the introduction of private capital played a role. Capital intensive industries are mainly distributed in the basic industries and heavy industries, and their development requires substantial capital investment, with the amount of fixed capital and current capital occupied by each worker being higher; further, foreign capital does not have capital advantages. Therefore, it is difficult to introduce foreign capital to improve the efficiency of enterprises. ③In the technology intensive industries, the efficiency of the three types of mixed ownership enterprises is significantly higher than the state-owned enterprises, and the coefficient of the mixed form containing foreign capital is greater, which shows that the technical advantages of foreign capital have been fully exploited, and the efficiency of state-owned enterprises raised by the introduction of foreign capital is the most obvious.

**Table 3:** The regression results of ownership structure on enterprise efficiency.

Explanatory variable	Full sample	Labor intensive	Capital intensive	Technology intensive
	TFP	TFP	TFP	TFP
**state-owned + private**	0.015**	0.027	0.029***	0.023*
	(2.18)	(1.60)	(2.79)	(1.89)
**state-owned + foreign**	0.028*	0.045	0.014	0.065**
	(1.81)	(1.36)	(0.50)	(2.12)
**state-owned + private + foreign**	0.067***	0.065*	0.122***	0.070**
	(3.70)	(1.71)	(3.94)	(2.16)
**size**	0.242***	0.167***	0.008***	0.008***
	(60.33)	(16.27)	(7.29)	(7.14)
**age**	-0.002***	-0.001	0.001	-0.001***
	(-6.11)	(-0.96)	(1.36)	(-2.60)
**lev**	-0.097***	-0.113***	-0.128***	-0.098***
	(-11.46)	(-5.99)	(-8.87)	(-6.26)
**fae**	-0.639***	-0.705***	-0.686***	-0.637***
	(-42.40)	(-19.43)	(-28.43)	(-23.21)
**sub**	-0.601***	-0.462**	-0.772***	-0.406***
	(-10.30)	(-2.08)	(-6.31)	(-5.91)
**year**	control	control	control	control
**pro**	control	control	control	control
**Constant term**	6.874***	7.365***	7.572***	6.488***
	(40.18)	(9.04)	(12.81)	(15.80)
**Number of samples**	259835	55172	97751	82421
**R-squared**	0.294	0.141	0.092	0.094

Note

* means significant at the 10% level

* * indicates significant at the level 5%

* * * indicates significant at the 1% level; t statistic value in parentheses.

By comparing the efficiency of the three forms of mixed ownership enterprises and state-owned enterprises, it can be found that mixed ownership is a more effective form of enterprise organization. The empirical evidence also shows that under mixed ownership, when non-state capital is involved in the operation of state-owned enterprises, different types of capital can complement and promote each other. The heterogeneous ownership structure has the supervision and incentive effect on the operation and management of the enterprise.

## The non-linear influence of heterogeneous ownership balance on the efficiency of mixed-ownership enterprises

To further verify the non-linear effect of the heterogeneous ownership balance on the enterprise's efficiency, this paper adds the first and the quadratic terms of the heterogeneous ownership balance degree to the model, and the regression models are models (2), (3), and (4); the regression results are shown in Table 4.

**Table 4:** The regression results of the effect of heterogeneous ownership balance degree on the efficiency of mixed ownership enterprises.

Explanatory variable	Model (2)	Model (3)	Model (4)
	TFP	TFP	TFP
**|A-B|**	-0.028*		-0.157***
	(-1.69)		(-2.78)
**|A-B|**^**2**^		-0.015	0.137**
		(-0.92)	(2.39)
**size**	0.319***	0.319***	0.319***
	(41.63)	(41.58)	(41.50)
**age**	-0.001	-0.001	-0.001
	(-0.60)	(-0.62)	(-0.60)
**lev**	-0.122***	-0.121***	-0.121***
	(-7.29)	(-7.27)	(-7.25)
**fae**	-0.694***	-0.694***	-0.694***
	(-25.18)	(-25.19)	(-25.18)
**sub**	-0.217**	-0.217**	-0.217**
	(-2.01)	(-2.01)	(-2.01)
**year**	control	control	control
**ind**	control	control	control
**pro**	control	control	control
**Constant term**	3.0744***	3.0717***	3.091***
	(0.3880)	(0.3881)	(0.3881)
**Sample size**	109210	109210	109210
**R-squared**	0.261	0.261	0.259

Note

* means significant at the 10% level

* * indicates significant at the level 5%

* * * indicates significant at the 1% level; t statistic value in parenthese.

The size of the heterogeneous ownership balance degree index set in this paper is inversely proportional to the degree of heterogeneous ownership balance in the mixed ownership. If the state-owned enterprises do not contain heterogeneous shareholders, that is, there is no heterogeneous ownership balance, according to the setting of the heterogeneous ownership balance degree index in this paper, the heterogeneous ownership balance degree in the state-owned enterprise is 1. With the introduction of non-state-owned shareholders in state-owned enterprises, the effect of heterogeneous ownership balance begins to increase, and the corresponding heterogeneous ownership balance degree begins to decrease from 1. When the holdings ratio of state-owned shareholders and non-state-owned shareholders is equal, the heterogeneous ownership balance degree is 0, which indicates that the effect of heterogeneous ownership balance is the strongest.

**Fig 1:**
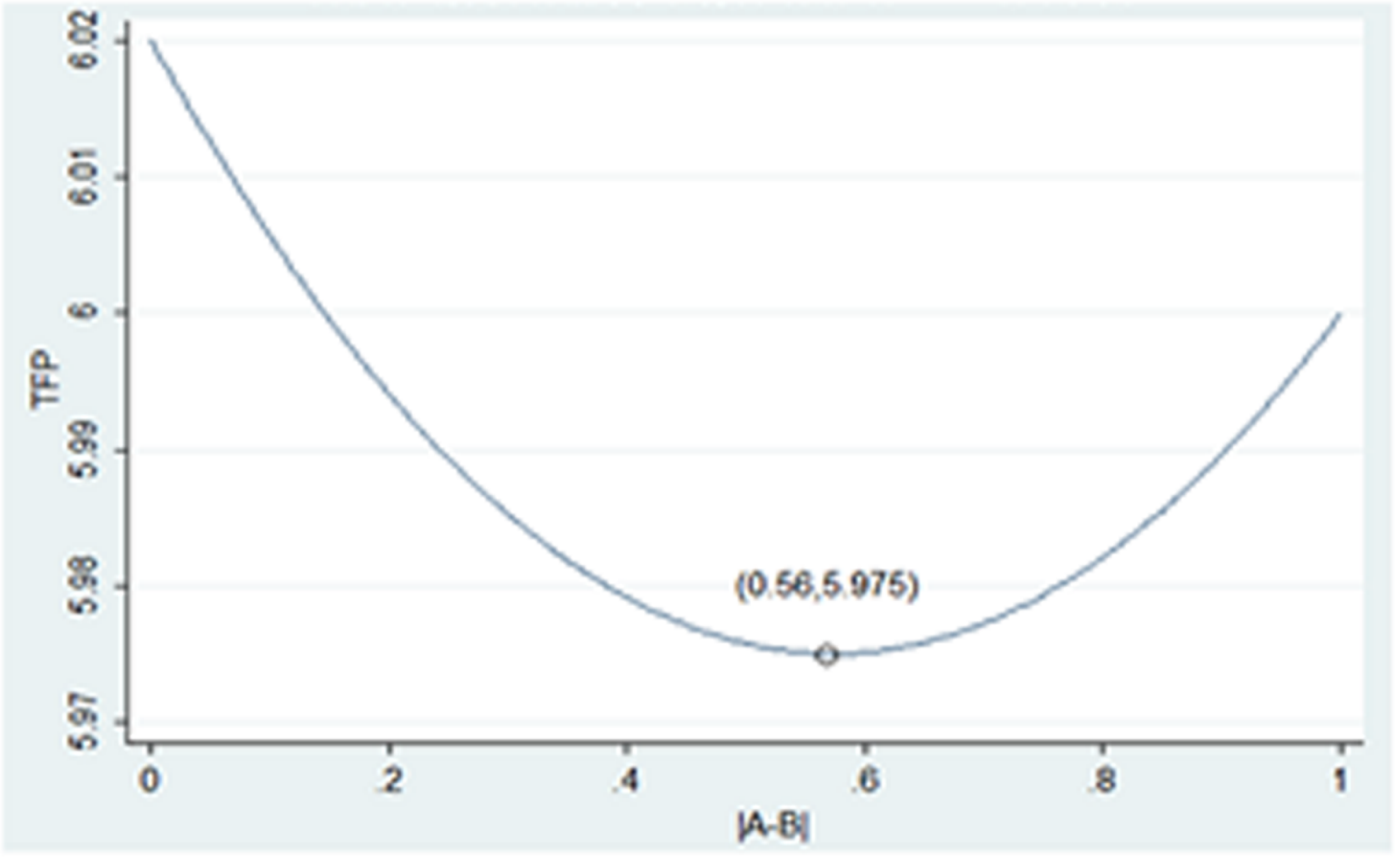
The relationship between heterogeneous ownership balance degree and the efficiency of mixed ownership enterprises.

In the regression results of the three types of models in Table 4, the coefficient of heterogeneous ownership balance degree in model (2) is significantly negative at the level of 10%, and the coefficient of the quadratic terms of the heterogeneous ownership balance degree in the model (3) is positive but not significant; model (4) also incorporates the heterogeneous ownership balance degree and its quadratic terms. The result is that the primary coefficient is significantly negative at the 1% level, and the quadratic coefficient is significantly positive at the 5% level. This shows that there is a U-type relationship between enterprise efficiency and heterogeneous ownership balance degree. To draw this curve more clearly, we bring the mean and constant terms of each control variable into model (4) to obtain Eq (5) and make the following Fig 1.

$$TFP=0.137{\left|A-B\right|}^{2}-0.157|A-B|+6.02$$ (5)

The following is to try to use the existing theory to explain this U-type relationship: ①In the process of heterogeneous ownership balance degree decreasing from 0.56 to 0, the proportion of competing shareholders in heterogeneous ownership enterprises is gradually increasing, and the degree of ownership balance of the competitive shareholders to the controlling shareholders is gradually becoming stronger; they have the motivation to fully exercise their supervisory power, which has played an effective supervisory role in the management. At the same time, the cost and risk of the controlling shareholder of the implementation of the "tunneling" behavior is increasing gradually, thus weakening the "tunnel effect"; the efficiency of enterprises has been improved under the combined effect of enhanced "supervisory effect" and weakened "tunneling effect". ②In the process of increasing heterogeneous ownership balance degree from 0.56 to 1, the intensity of ownership balance between the two types of shareholders is gradually declining, and the controlling shareholder's control over the enterprise is increasing constantly. At the same time, the controlling shareholder can obtain increasingly more dividends from the promotion of enterprise value, and the willingness to "empty out" gradually decreases, so the efficiency of the enterprise begins to increase. ③When the heterogeneous ownership balance degree is approximately 0.56, the two shareholders’ shareholding ratio is approximately 0.78 and 0.22, although there exists a competitive shareholder, but its shareholding is limited. Although the competitive shareholder is willing to constrain the controlling shareholder, it will more likely have a conflict of interest with them and lead to the lowest of the enterprise’s efficiency. Thus, it is the gray area of the influence of the heterogeneous ownership balance degree on efficiency.

The results of the previous analysis show that the effect of heterogeneous ownership balance on enterprise efficiency is the non-linear U-type relationship, and there is a "gray region" of ownership balance. Despite there being a certain balance between the two types of shareholders, due to the limited shareholding of small shareholders, this gray region can not only cannot serve as a good constraint on the big shareholders but will also create conflicts among shareholders and increase the cost of corporate governance. To further determine the "gray area" of the ownership balance in the U-type relationship, this paper divides the sample into multiple intervals according to the heterogeneous ownership balance degree (| A-B |), and the fixed efficiency model is adopted to do a regression without consideration of the quadratic terms of the heterogeneous ownership balance degree. The specific regression coefficients and corresponding statistical significance are shown in Table 5.

**Table 5:** The regression results between partitions.

Interval	Coefficient	The value of T	Interval	Coefficient	The value of T
0–0.1	-0.6265	-0.84	0.5–0.6	0.3018	0.32
0.1–0.2	-0.3038	-0.33	0.6–0.7	0.022	-0.03
0.2–0.3	-1.3639*	-1.79	0.7–0.8	2.0182**	2.59
0.3–0.4	-1.5341*	-1.92	0.8–0.9	1.6531**	2.1
0.4–0.5	-1.6291*	-1.8	0.9–1	-0.6834	-0.81

Note

* means significant at the 10% level

* * indicates significant at the level 5%; t statistic value in parentheses.

The results in Table 5 show that the coefficient in the interval is negative but not significant where the heterogeneous ownership balance degree is greater than 0 and less than 0.2; in the interval where heterogeneous ownership balance degree is greater than 0.2 and less than 0.5, the coefficients are significantly negative (below 10%); and when the heterogeneous ownership balance degree is greater than 0.5 and less than 0.6, the coefficient begins to become positive but not significant. When the heterogeneous ownership balance degree is greater than 0.7 and less than 0.9, the coefficients are significantly positive (below 5%). Thus, it can be inferred that the "gray area" of ownership balance degree of the influence of heterogeneous ownership balance degree on enterprise efficiency is between 0.2 to 0.5.

In view of the above measurement results, this paper explains that when the heterogeneous ownership balance degree is in the range of [0.2, 0.5], the shareholdings of the two types of shareholders are 40% to 25% and 60% to 75%. At the same time, although there is a heterogeneous ownership balance of shareholders in the mixed ownership enterprises, the number of its holdings rather than the controlling shareholders have a certain gap, which leads to the lack of ability to balance shareholder participation in management and easily leads to conflicts of interest with controlling shareholders; furthermore, the "opinion divergence effect" is significant. At the same time, because the number of holdings of the controlling shareholder is not absolute enough, the loss caused by the decline in the efficiency of the enterprise is still partially distributed to the other shareholders, so the shareholders can pursue personal interests by tunneling the enterprise. Therefore, heterogeneous ownership balance is negatively correlated with enterprise efficiency when the heterogeneous ownership balance degree is in the range of [0.2, 0.5]. When the heterogeneous ownership balance degree is in the range of [0.7, 0.9], the shareholding gap of the two types of shareholders is very large, and the conflict of the two types of shareholders is weakened. At the same time, the controlling shareholders can harvest most of the benefits of the promotion of enterprise’s value, and the willingness to "hollow out" the business will be reduced.

## Research conclusions and implications

Using the data of Chinese industrial enterprise database, this paper studies the difference in efficiency between mixed ownership enterprises and state-owned enterprises as well as the non-linear effect of heterogeneous ownership balance on the efficiency of mixed ownership enterprises. The empirical evidence shows that the efficiency of the three forms of mixed ownership enterprises is higher than that of state-owned enterprises. The more diversified the form is, the higher the efficiency becomes, and the introduction of private capital or foreign capital by different industries will have different effects. There is a non-linear U-shaped relationship between the heterogeneous ownership balance and enterprise efficiency. With the increase in the heterogeneous ownership balance degree, the efficiency of enterprises decreases and then increases. Higher and lower heterogeneous ownership balance degrees will promote enterprise efficiency; when the ownership balance degree is in the range of [0.2, 0.5], an increase of the balance degree will lead to a decline of enterprise efficiency.

With the ever-changing market environment, the promotion of mixed ownership reform, the development of mixed ownership economy, the establishment of a diversified ownership structure and ultimately the realization of an ideal hybrid ownership structure needs to be explored step by step by the enterprise in the market competition practice. The conclusion of this paper has important policy implications for the mixed ownership reform of state-owned enterprises. First, the empirical results show that the efficiency of mixed ownership enterprises is higher than the efficiency of state-owned enterprises, and the diversified ownership structure is conducive to the improvement of enterprise efficiency. It proves that the mixed ownership reform can become one of the important ways to reform the state-owned enterprises in our country. Second, due to the different nature of capital in different industries have different effects, and each enterprise is faced with different competitive characteristics because of its different market environment, and it must have a different optimal ownership structure. Therefore, with mixed ownership, state-owned enterprises must tailor their own characteristics to the introduction of non-state capital and the construction of optimal mixed ownership structure. Third, the non-linear U-shaped relationship between the heterogeneous ownership balance and enterprise efficiency shows that in the case of mixed ownership reform, it is necessary to give up linear thinking and analyze the specific problems from the enterprise itself as well as design a reasonable mixture proportion of heterogeneous equity to improve the efficiency of the company. we need a "bottom-up" model rather than the "one size fits all" model to fully respect enterprises and the independent decision-making power of entrepreneurs, and stimulate their subjective initiative and creativity.

## Supporting information

S1 Original Data The original data are available from the database data of Chinese industrial enterprises from 2000 to 2007 to replicate our findings in this article. (ZIP)
